# Levothyroxine poisoning in children: a multicenter study from Türkiye

**DOI:** 10.55730/1300-0144.6152

**Published:** 2025-12-18

**Authors:** Kübra ŞEN KÜÇÜK, Şule DEMİR, Reyhan DEVECİ SEVİM, Fatma AKGÜL, Gülşen YALÇIN, Öznur ESER, Alkan BAL, Elif ERGİN, Sercan ÖZTÜRK, Ahmet ANIK

**Affiliations:** 1 Division of Pediatric Endocrinology, Department of Pediatrics, Faculty of Medicine, Aydın Adnan Menderes University, Aydın, Turkiye; 2 Division of Pediatric Emergency, Department of Pediatrics, Faculty of Medicine, Aydın Adnan Menderes University, Aydın, Turkiye; 3 Division of Pediatric Emergency, Department of Pediatrics, Dr. Behcet Uz Pediatric Diseases and Surgery Training and Research Hospital, İzmir, Turkiye; 4 Division of Pediatric Emergency, Department of Pediatrics, Buca Seyfi Demirsoy Training and Research Hospital, İzmir, Turkiye; 5 Division of Pediatric Emergency, Department of Pediatrics, Faculty of Medicine, Dokuz Eylul University, İzmir, Turkiye; 6 Division of Pediatric Emergency, Department of Pediatrics, Faculty of Medicine, Celal Bayar University, Manisa, Turkiye; 7 Division of Pediatric Emergency, Department of Pediatrics, Faculty of Medicine, Ege University, İzmir, Turkiye; 8 Department of Pediatrics, Faculty of Medicine, Aydın Adnan Menderes University, Aydın, Turkiye

**Keywords:** Hypothyroidism, levothyroxine poisoning, multiple drug poisoning

## Abstract

**Background/aim:**

This study aimed to investigate the clinical features, laboratory findings, treatment approaches, and follow-up strategies employed in the management of pediatric patients with levothyroxine poisoning.

**Materials and methods:**

This multicenter retrospective study included patients aged ≤18 years who presented with acute, single-episode levothyroxine ingestion to the pediatric emergency departments of six tertiary care centers between 2010 and 2023. Clinical data, laboratory findings, symptom onset and severity, and treatment modalities were extracted from medical records. Thyroid function tests (TFTs) were interpreted according to the age-specific reference ranges of each center. Treatment decisions, including gastric decontamination and pharmacologic therapy, were evaluated based on documented clinical indications.

**Results:**

A total of 45 patients were included in the study. The median age was 3.83 years (interquartile range [IQR]: 2.2–6.1). Levothyroxine ingestion was accidental in 80% (n = 36) of cases and intentional (suicidal) in 20% (n = 9). The median ages for accidental and suicidal ingestions were 3.0 years (IQR: 2.0–4.2) and 15.0 years (IQR: 13.7–16.9), respectively (p < 0.001). The median total ingested levothyroxine dose was 500 μg (IQR: 212.5–1112.5). Symptoms were reported in 33.3% (n = 15) of patients, with tachycardia being the most common (100%, n = 15), followed by hypertension (26.7%, n = 4) and agitation (20.0%, n = 3). The median levothyroxine dose among symptomatic patients was 1100 μg (IQR: 450–2250), significantly higher than that of asymptomatic patients 312.5 μg (IQR: 187.5–750) (p = 0.003). TFTs were abnormal in 51.1% (n = 23) of patients. Treatment was initiated in 55.6% (n = 25) of patients; activated charcoal was administered to 51.1% (n = 23), and gastric lavage to 26.7% (n = 12). Among the three patients with moderate symptoms, one received propranolol, methimazole, and corticosteroids, whereas the other two were treated with propranolol alone. An asymptomatic patient with markedly abnormal TFTs received corticosteroids. No fatal outcomes were reported.

**Conclusion:**

Levothyroxine poisoning in children is generally asymptomatic. When symptoms do occur, they are typically mild to moderate in severity, and the overall prognosis is favorable.

## 1. Introduction

Levothyroxine (LT4) ingestion is relatively uncommon among children. However, compared with adults, levothyroxine ingestion occurs more frequently in children and generally follows a more benign clinical course [1]. Although children often exhibit higher serum LT4 levels than adults, severe symptoms are less frequently observed in this group [2]. This apparent increased tolerance to LT4 in children may be attributed to the higher hepatic metabolic capacity characteristic of childhood [3].

In a 2015 study from the United States, a total of 9325 cases of thyroid hormone poisoning were reported to the American Association of Poison Control Centers (AAPCC). Of these cases, 50% involved children under 5 years of age, 10% were in those aged 6–19 years, and 40% occurred in individuals older than 19 years [4]. Massive LT4 overdoses are usually accidental, most often occurring in young children and, less commonly, in adolescents. In contrast, intentional overdoses are more frequently observed in adolescents and adults, often associated with suicidal intent, weight-loss attempts, or other undisclosed motives [1].^1^

In children and adolescents, the clinical course is typically mild. Common signs and symptoms include fever, flushing, palpitations, excessive sweating, tremor, nervousness, insomnia, tachycardia, hypertension, and loose stools. Rarely, more severe delayed-onset complications may occur, including coma, convulsions, acute psychosis, thyroid storm, and cardiac arrhythmias. Symptoms may be delayed for up to 11 days following an LT4 overdose, consistent with its long half-life of approximately 7 days [5].

The management of thyroid hormone ingestion remains a subject of debate [5]. The initial absence of symptoms, combined with considerable interindividual variability in clinical response, complicates the assessment of treatment effectiveness in such cases [3]. Although pediatric LT4 ingestion is frequently reported to poison control centers, real-world multicenter data describing symptom onset, severity patterns, and variations in clinical management remain scarce. The existing evidence is primarily derived from case reports and small single-center series, with substantial variation in treatment approaches across institutions.

Therefore, comprehensive observational data are needed to better characterize current clinical practice and outcomes in children with LT4 ingestion. In this multicenter study, we aimed to comprehensively evaluate the clinical presentation, management strategies, and outcomes of pediatric patients with LT4 intoxication.

## 2. Materials and methods

This multicenter retrospective study included pediatric patients who presented with LT4 ingestion to six tertiary pediatric centers in Türkiye between January 2010 and December 2023. The participating centers were Aydın Adnan Menderes University Faculty of Medicine, Dokuz Eylül University Faculty of Medicine, Ege University Faculty of Medicine, Celal Bayar University Faculty of Medicine, University of Health Sciences Dr. Behçet Uz Child Health and Surgery Training and Research Hospital, and Buca Seyfi Demirsoy Training and Research Hospital. Ethical approval was obtained from the institutional review boards of all participating centers. As this was a retrospective study, the requirement for informed consent was waived.

### 2.1. Data collection and management

The collected data for all patients included age, sex, reason for ingestion, presence of comorbidities (e.g., hypothyroidism), concurrent medication use, estimated time from ingestion to emergency department presentation, LT4 dose ingested, and clinical findings and vital signs at presentation and during follow-up.

Laboratory evaluations included thyroid-stimulating hormone (TSH), free thyroxine (fT4), and free triiodothyronine (fT3), as well as complete blood count parameters (hemoglobin, hematocrit, leukocyte, and platelet counts), renal function tests (blood urea nitrogen and creatinine), and liver enzymes (aspartate aminotransferase and alanine aminotransferase). Thyroid function tests (TFTs) were performed in the biochemistry laboratories of each participating center using automated chemiluminescent immunoassay analyzers. Because assay platforms and reference ranges differed slightly among centers, results were interpreted according to the age-specific reference intervals of the respective laboratories. To minimize intercenter variability, the analyses focused on categorical interpretation (normal versus abnormal) rather than on absolute hormone concentrations. An abnormal TFT was defined as elevated fT4 and/or fT3 levels, with TSH being low, suppressed, or within the normal range, according to the age-specific reference ranges of each laboratory. Because reference intervals differed across centers, uniform normal ranges for TSH, fT4, and fT3 could not be established. Additionally, the length of hospital stay and duration of outpatient follow-up were recorded.

### 2.2. Clinical symptom assessment

Clinical symptoms were retrospectively assessed based on documentation from the initial pediatric emergency department evaluation and progress notes recorded throughout hospitalization. Delayed-onset symptoms were additionally identified through inpatient observation and, when available, scheduled outpatient follow-up visits after discharge. Both the presence and timing of symptom onset were recorded.

### 2.3. Definition of clinical symptoms

Standardized criteria were applied to classify clinical manifestations as follows:

- **Tachycardia:** Heart rate above the 99th percentile for age [6].
- **Hypertension:** Systolic and/or diastolic blood pressure above the 95th percentile for age, sex, and height [7].
- **Agitation:** Clinically documented excessive motor activity or behavioral disturbance.
- **Restlessness:** A persistent inability to remain still, as clinically observed and documented during patient assessment.
- **Headache:** Patient- or caregiver-reported head pain documented in the medical record.
- **Confusion:** Altered mental status characterized by disorientation, impaired attention, or slowed responses.

### 2.4. Classification of symptom severity

Symptom severity was categorized as follows:

- **Mild:** Self-limited symptoms without hemodynamic compromise.
- **Moderate:** Symptoms requiring close clinical observation or pharmacologic intervention at the clinician’s discretion, including but not limited to tachycardia, hypertension, agitation, or restlessness.
- **Severe:** Life-threatening manifestations, such as arrhythmias, seizures, or thyroid storm.

### 2.5. Treatment protocol and clinical management

Treatment-related data were retrospectively extracted from patient medical records. Due to the absence of a standardized national protocol for LT4 intoxication management and the retrospective nature of the study, documentation of treatment indications was occasionally incomplete. The treatment criteria were not defined a priori; instead, they were reconstructed retrospectively based on consistent documentation patterns observed across patient records in all participating centers. Treatment decisions were made by pediatric emergency physicians and/or pediatric endocrinologists based on the clinical presentation, estimated ingested dose, elapsed time since ingestion, TFT results, and—at several centers—the recommendations of the National Poison Control Hotline (UZEM, Türkiye), as documented in the medical records.

Gastric decontamination was performed at the discretion of the treating clinicians and involved the administration of activated charcoal (1 g/kg) and/or gastric lavage. Based on the retrospective chart review, gastric decontamination was most commonly applied in patients who:

- presented within 4 h after LT4 ingestion,
- exhibited symptoms upon admission,
- received a recommendation from UZEM.

Although activated charcoal is most effective when administered within the 1st h, the exact time of ingestion was often uncertain, particularly among children presenting 1–4 h after exposure. For this reason, and in accordance with UZEM recommendations, gastric decontamination was occasionally performed beyond the optimal 1 h window.

Pharmacologic treatments were selectively administered according to symptom severity and clinician judgment:

- Propranolol was initiated in patients with persistent tachycardia or hypertension requiring symptomatic control.
- Antithyroid drugs (e.g., methimazole) were used in a limited number of patients with markedly elevated thyroid hormone levels and/or moderate symptoms.
- Corticosteroids were administered in selected cases to inhibit the peripheral conversion of fT4 to fT3 or in patients whose symptoms were classified as moderate.

### 2.6. Eligibility criteria

#### 2.6.1. Inclusion criteria

1. Patients aged ≤18 years.
2. Presentation to the pediatric emergency departments of participating centers with acute, single-episode LT4 ingestion, verified by at least one of the following:
  - patient or caregiver report,
  - pill count discrepancy,
  - identification of LT4 medication belonging to the patient or a family member,
  - confirmed access to (e.g., obtaining the medication from a pharmacy without a prescription).
3. Availability of at least one complete set of TFTs (TSH, fT4, and fT3).
4. Availability of complete and accessible hospital records.

#### 2.6.2. Exclusion criteria

1. Patients aged >18 years.
2. Patients with missing or inaccessible medical records.
3. Patients with chronic supratherapeutic LT4 use (e.g., for weight-loss purposes).
4. Patients with chronic diseases that may mimic hyperthyroidism symptoms (e.g., cardiac arrhythmia, primary hypertension, pheochromocytoma).
5. Patients with known primary hyperthyroidism (e.g., Graves’ disease, toxic nodular goiter).
6. Patients with a history of medication-induced thyrotoxicosis without LT4 exposure (e.g., amiodarone).

### 2.7. Statistical analysis

Data analysis was performed using SPSS Statistics for Windows, version 21.0 (IBM Corp., Armonk, NY, USA). Descriptive statistics were expressed as mean ± standard deviation for normally distributed variables and as median (25th–75th percentile) for nonnormally distributed variables. Categorical variables were compared using the chi-square test. The Student’s t-test was used to compare continuous variables between independent groups with parametric distributions, whereas the Mann–Whitney U test was applied for nonparametric distributions. For paired continuous variables, the paired t-test was employed for parametric data, and the Wilcoxon signed-rank test for nonparametric data. Correlation analyses were performed using Pearson’s correlation coefficient for parametric data and Spearman’s rank correlation coefficient for nonparametric data. Multivariate logistic regression analysis using the backward Wald method was performed to identify independent predictors of outcomes. A p-value <0.05 was considered statistically significant.

## 3. Results

**Figure 1:**
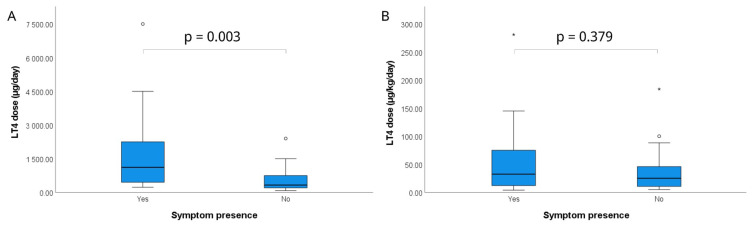
(A) Daily LT4 dose (μg/day) and (B) weight-adjusted LT4 dose (μg/kg/day) in symptomatic and asymptomatic patients. The total daily LT4 dose was significantly higher in symptomatic patients (p = 0.003).

A total of 45 children (62.2%, n = 28 females) were enrolled in the study. LT4 ingestion was accidental in 80% (n = 36) of cases and intentional (suicidal) in 20% (n = 9). The median age was 3.83 (2.2–6.1) years (Table 1). The median ages for accidental and intentional (suicidal) ingestion were 3.0 (2.0–4.2) years and 15.0 (13.7–16.9) years, respectively (p < 0.001). Comorbidities were present in 24.4% (n = 11) of patients, most commonly hypothyroidism (72.7%, n = 8), followed by substance dependence, obsessive–compulsive disorder, and developmental delay (each 9.1%, n = 1). All eight patients with hypothyroidism were receiving LT4 therapy and were intoxicated with their own medication, whereas the remaining patients (n = 37) ingested LT4 belonging to a family member. A total of nine patients (20%) were concurrently taking multiple medications (Table 1). Multiple drug ingestion occurred in 66.7% (n = 6) of patients with intentional (suicidal) LT4 ingestion, compared with 8.3% (n = 3) among those with accidental ingestion (p < 0.01). The median LT4 dose ingested was 500 (212.5–1112.5) μg/day and 27 (10.55–53.25) μg/kg/day. Overall, 66.7% (n = 30) of patients were asymptomatic, whereas 33.3% (n = 15) exhibited symptoms. The most common symptom was tachycardia (100%, n = 15), followed by hypertension (26.7%, n = 4), agitation (20%, n = 3), restlessness (6.7%, n = 1), headache (6.7%, n = 1), and confusion (6.7%, n = 1) (Table 1). Symptom severity was mild in 73.3% of cases (n = 11) and moderate in 26.7% (n = 4); no instances of severe toxicity were observed. Among the 15 symptomatic patients, 14 exhibited symptoms at presentation to the pediatric emergency department. These patients presented within 0–1 h (21.4%, n = 3), 1–4 h (71.4%, n = 10), or 4–8 h (7.2%, n = 1) following ingestion and were therefore classified as having acute-onset symptoms. A patient developed tachycardia 24 h after admission to the pediatric ward, consistent with delayed-onset presentation (Table 2). Later presentations were observed in two asymptomatic patients, occurring 12–24 h and 24–48 h after ingestion. Overall, there was no statistically significant difference in time to presentation between symptomatic and asymptomatic patients (p > 0.05). The median LT4 dose among symptomatic patients was 1100 (450–2250) μg/day, significantly higher than that of asymptomatic patients 312.5 (187.5–750) μg/day (p = 0.003) (Figure 1A). However, the median LT4 dose was 35 (12–75) μg/kg/day in symptomatic patients and 25 (10–48.4) μg/kg/day in asymptomatic patients (p = 0.37) (Figure 1B). Symptom prevalence was higher among patients taking multiple medications (66.7%, n = 6) than among those not taking multiple medications (25%, n = 9) (p = 0.042). The characteristics of the 15 symptomatic patients are summarized in Table 2.

**Figure 2:**
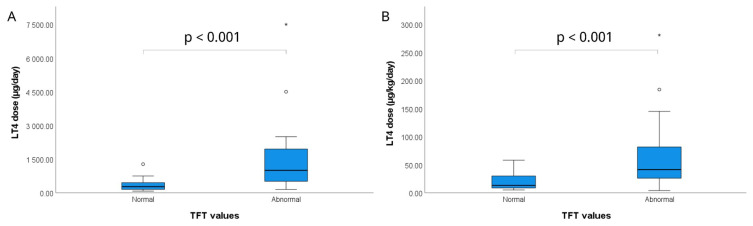
(A) Daily LT4 dose (μg/day) and (B) weight-adjusted LT4 dose (μg/kg/day) in patients with normal and abnormal thyroid function tests (TFTs). Both dose types were significantly higher in patients with abnormal TFTs (p < 0.001 for both comparisons).

Upon admission, respiratory rate, body temperature, hemoglobin, white blood cell, and platelet counts, as well as blood urea nitrogen, creatinine, aspartate aminotransferase, and alanine aminotransferase levels, were within normal reference ranges in all patients and remained stable throughout the follow-up period. TFTs were abnormal in 51.1% (n = 23) of patients (Table 1). TFTs were obtained for all patients upon presentation to the pediatric emergency department. However, the interval between LT4 ingestion and hospital admission varied considerably among patients; therefore, initial TFTs were not performed at a standardized postingestion time point. Similarly, follow-up TFTs obtained during hospitalization or outpatient follow-up were measured at variable intervals, reflecting clinical necessity, physician discretion, and intercenter variations in monitoring practices. Consequently, the temporal trends of thyroid hormone levels could not be uniformly assessed across all patients. The median serum TSH, fT4, and fT3 levels at admission were 1.4 (0.9–2.1) mIU/L, 1.4 (1.1–2.9) ng/dL, and 3.5 (3.1–4.8) pg/mL, respectively (Table 3). The absolute LT4 dose was significantly greater in the abnormal TFT group, with a median of 1000 (512.5–1950) μg/day, compared with 275 (150–450) μg/day in patients with normal TFTs (p < 0.001) (Figure 2A). Likewise, the weight-adjusted LT4 dose was markedly higher in patients with abnormal TFTs, with a median of 41.3 (26–81.6) μg/kg/day, compared with 13 (8.69–30) μg/kg/day in those with normal TFTs (p < 0.001) (Figure 2B). Abnormal TFTs were observed in 73.3% (n = 11) of symptomatic patients and 40% (n = 12) of asymptomatic patients (p = 0.036) (Table 2).

In the backward Wald logistic regression analysis, the initially included variables—LT4 dose (μg/day), TFT abnormality, multiple drug ingestion, and cause of ingestion—were assessed. During the stepwise elimination process, multiple drug ingestion and TFT abnormality were sequentially removed from the model. In the final model, LT4 dose and suicidal ingestion remained significant predictors of symptomatic presentation. Higher LT4 doses (μg/day) were associated with a modest yet statistically significant increase in the odds of symptomatic presentation (OR = 1.001; p = 0.045). Suicidal ingestion, however, was the strongest predictor, conferring an approximately 33-fold higher risk of symptomatic presentation (OR = 33.36; p = 0.004) (Table 4).

Treatment was initiated in 55.6% (n = 25) of patients. Activated charcoal was administered to 51.1% (n = 23) of patients, whereas gastric lavage was performed in 26.7% (n = 12) (Table 1). Among the 38 patients who presented within 4 h of ingestion, 55.3% (n = 21) underwent gastric decontamination. Symptomatic patients were significantly more likely to undergo decontamination than asymptomatic patients (84.6%, n = 11 versus 40%, n = 10). Additionally, two asymptomatic patients who presented between 4 and 8 h postingestion underwent decontamination upon the recommendation of UZEM. Pharmacologic therapy was initiated in four patients (Table 1). A patient who ingested 2100 μg/day of LT4 presented with tachycardia, hypertension, restlessness, agitation, and abnormal TFTs. He received propranolol for cardiovascular symptoms, methimazole for markedly elevated thyroid hormone levels, and methylprednisolone to inhibit peripheral T4-to-T3 conversion. Patients who ingested 1125 μg/day and 600 μg/day of LT4, respectively, were treated with propranolol after developing tachycardia—acute in one case and delayed in the other (Table 2). Additionally, one asymptomatic patient with abnormal TFTs after ingesting 525 μg/day received a short course of oral dexamethasone to inhibit peripheral conversion of T4 to T3 (Table 1).

No serious complications or fatalities occurred, and none of the patients required plasma exchange or hemodialysis. The median duration of hospitalization was 24 (12–48) h, and the median duration of outpatient follow-up was 3 (0–14) days. The median time to TFT normalization was 0.5 (0–7) days (Table 1). Although gastric decontamination was not guided by TFT results, retrospective analysis revealed no statistically significant difference in the time to TFT normalization between patients with abnormal TFTs who underwent decontamination and those who did not (p = 0.720).

## 4. Discussion

This multicenter study demonstrates that LT4 poisoning in the pediatric population generally follows a benign clinical course, with most manifestations being mild to moderate in severity. While these findings align with previously published pediatric toxicology data, they provide additional insight into the dose–response relationship, temporal patterns of symptom onset, and intercenter variability in management practices.

In our cohort, 80% of LT4 ingestions were accidental, and these patients were significantly younger than those with intentional ingestions. This age-related distribution closely mirrors the epidemiological trends reported by the AAPCC [4]. Previous studies have consistently shown that accidental LT4 ingestion is the predominant mechanism in young children, whereas intentional overdoses—including suicide attempts—occur more frequently in adolescents and adults [3,8]. Our findings are consistent with this established pattern and further reinforce the current epidemiological understanding of LT4 intoxication in the pediatric population.

Several reviews of acute pediatric LT4 poisoning have consistently reported that most affected children either remain asymptomatic or experience only mild, self-limiting symptoms. Notably, even relatively high ingested doses—up to 7.5 mg or 13 mg—have been reported to cause no clinical manifestations in certain cases [1,2,9–12]. In contrast, isolated reports have described severe manifestations such as seizures following LT4 ingestions of 3.6 mg and 18 mg, respectively, highlighting that significant toxicity—although rare—can occur across a wide dose range [13,14]. Consistent with these observations, our cohort included a child who ingested 7.5 mg of LT4 and, despite concomitant multiple-drug ingestion, developed only moderate symptoms and achieved complete recovery with supportive management.

Taken together, these studies indicate that a consistent and predictable dose–response relationship in LT4 poisoning cannot be reliably established. Several authors have proposed practical dose thresholds: the AAPCC recommends medical evaluation for suspected ingestions of 0.5–2 mg LT4 [15]; one study predicts clinically significant toxicity above 2–4 mg, particularly in older individuals with comorbidities [16]; Tsutaoka et al. reported that ingestions below 3 mg are generally asymptomatic [13]; and Tunget et al. suggested that ingestions below 5 mg typically cause only mild symptoms, making aggressive decontamination unnecessary in most cases [11]. Similarly, pediatric series involving even higher ingested doses have predominantly reported mild and transient clinical courses, underscoring the considerable interindividual variability in clinical response [9,12].

In our cohort, symptom development was clearly associated with the absolute LT4 dose, whereas no significant difference was observed when doses were adjusted for body weight. Nevertheless, a definitive threshold for symptom onset could not be established, suggesting that clinical expression is determined not only by the ingested dose but also by individual susceptibility and concomitant factors. Importantly, even relatively low doses may occasionally precipitate severe manifestations, making it difficult to predict which patients will become symptomatic [11]. This observation was further supported by our multivariable logistic regression analysis, which demonstrated that higher absolute LT4 doses were associated with a modest yet statistically significant increase in the likelihood of symptomatic presentation, although considerable interindividual variability persisted. Severe toxicity is observed less frequently following acute ingestion than with repeated exposure, and patients with acute ingestion generally have a more favorable prognosis [10]. Consistent with these findings, all patients in our study experienced acute overdoses, and the majority remained asymptomatic.

Delayed-onset manifestations of LT4 intoxication have been described in previous reports and are typically attributed to the gradual peripheral conversion of T4 to biologically active T3 [1,3,5,17–19]. However, our multicenter findings indicate that clinically significant symptoms in children generally appear early rather than late. Despite considerable variability in the interval between ingestion and medical evaluation, most symptomatic patients were already symptomatic upon initial presentation, and only one patient developed delayed tachycardia during hospitalization. These results suggest that although delayed symptoms are biologically plausible, their clinical significance in acute pediatric ingestions appears limited. Instead, interindividual susceptibility and the ingested dose appear to exert greater influence than the timing of presentation. Nonetheless, since delayed effects have been documented in some reports—particularly among children with markedly elevated initial hormone levels—expert recommendations continue to support short-term inpatient observation (3–5 days), followed by outpatient monitoring between days 3 and 10, when clinically indicated [5].

Children with abnormal TFTs had higher absolute and weight-adjusted LT4 doses, consistent with prior pediatric studies reporting that biochemical abnormalities are more likely at higher ingestion levels. However, clinical expression remained highly variable: some children exhibited only mild symptoms despite substantial hormone elevations, whereas others with comparable biochemical profiles were entirely asymptomatic [1,3,9]. In our cohort, abnormal TFTs were significantly more common among symptomatic patients, suggesting that biochemical derangements may contribute to symptom development in a subset of cases. Nonetheless, the substantial overlap between symptomatic and asymptomatic patients with abnormal TFTs indicates that neither hormone levels nor ingested dose alone reliably predict clinical severity, underscoring the multifactorial nature of LT4 toxicity.

Although our univariable analyses suggested that children who coingested additional medications were more likely to develop symptoms, this association did not remain significant in the multivariable model. A similar observation was reported in a previous study [3], which found a higher frequency of symptoms among children with multiple-drug ingestions. However, as most of these cases involved intentional overdoses, coingestion likely serves as a marker of higher-risk behavior rather than an independent determinant of toxicity. Likewise, in our cohort, most children who coingested other medications had intentional ingestions, and suicidal ingestion emerged as the strongest independent predictor of symptomatic presentation in the logistic regression analysis. Taken together, these findings suggest that the initial association between multiple-drug ingestion and symptom development likely reflects confounding by intent and dose rather than a true pharmacologic interaction.

Management strategies for pediatric LT4 intoxication vary considerably across studies, and no universally accepted threshold for treatment has been established. Because prophylactic antidotes or gastric decontamination have occasionally been employed even after relatively low ingestion doses, identifying which patients genuinely benefit from intervention remains challenging [11]. Consequently, many authors advocate a symptom-directed approach rather than treatment decisions based solely on the ingested dose. Within this framework, gastrointestinal decontamination, propranolol, antithyroid drugs, and corticosteroids are typically reserved for patients exhibiting overt thyrotoxic symptoms or markedly elevated hormone levels [9,11,18]. Treatment practices in our cohort were consistent with these recommendations: propranolol was administered for persistent tachycardia or hypertension, whereas methimazole and short-course corticosteroids were used selectively in patients with more pronounced biochemical abnormalities.

No standardized criteria have been established in the literature regarding the indications for gastrointestinal decontamination in LT4 intoxication. Reported thresholds vary considerably: while some authors consider decontamination unnecessary for ingestions below 0.5 mg, others recommend administering activated charcoal for doses exceeding 3–5 mg, particularly when given within the 1st h [11,12,19]. Gastric lavage is generally reserved for ingestions above 10 mg. It should also be noted that activated charcoal is not without risk, as it may cause vomiting or aspiration pneumonitis [13,19]. In our cohort, decisions regarding gastrointestinal decontamination were not guided by a clear dose-based threshold. The procedure was performed even in some children with low-dose ingestion and in those without coingested medications. Decisions were primarily driven by early presentation (within 4 h of ingestion), the presence of symptoms, and recommendations from UZEM.

No deaths related to acute LT4 overdose have been reported in either pediatric or adult populations [11], consistent with our observation of an overall benign course without severe symptoms or fatalities. The primary limitations of this study are its retrospective design and the nonstandardized timing of TFT assessments. Nonetheless, the relatively large sample size and multicenter design are notable strengths that enhance the generalizability of our findings.

In conclusion, our study reinforces the predominantly benign nature of pediatric LT4 intoxication, which can generally be managed effectively with supportive care alone. Most asymptomatic children—particularly those with accidental low-dose ingestions—can be safely monitored with short-term observation and generally do not require pharmacologic treatment. Gastric decontamination should be considered primarily for patients presenting within the first 4 h or those who exhibit early symptoms. Propranolol is appropriate for persistent tachycardia or hypertension, whereas antithyroid drugs or corticosteroids should be reserved for selected cases presenting with marked hormonal elevations or moderate symptoms. These practical recommendations may assist clinicians in making risk-based decisions regarding the evaluation and management of pediatric LT4 ingestion. Given the variability in biochemical and clinical responses and the lack of a reliable dose threshold for predicting symptoms, future prospective studies are warranted to refine monitoring protocols and establish evidence-based management guidelines.

## Figures and Tables

**Table 1:** Demographic and clinical characteristics of the patients (n = 45).

Characteristics	All patients % (n)

**Sex**	
Female	62.2 (28)
Male	37.8 (17)

**The ingested drug**	
Levothyroxine	97.8 (44)
Levothyroxine and triiodothyronine	2.2 (1)

**Cause of ingestion**	
Accidental	80.0 (36)
Suicidal	20.0 (9)

**Age (year)** * †	3.8 (2.2–6.1)
Accidental ingestion–median age*	3.0 (2.0–4.2)
Suicidal ingestion–median age *	15.0 (13.7–16.9)

**Comorbidity**	24.0 (11)
Hypothyroidism	72.7 (8)
Substance dependence	9.1 (1)
Obsessive-compulsive disorder	9.1 (1)
Developmental delay	9.1 (1)

**Source of ingested medication**	
Family member	82.3 (37)
Their own medication	17.7 (8)

**Multiple drugs** ‡	20.0 (9)
Accidental	8.3 (3)
Suicidal	66.7 (6)

**Time from ingestion to presentation (hours)**	
0–1	17.8 (8)
1–4	66.7 (30)
4–8	11.1 (5)
12–24	2.2 (1)
24–48	2.2 (1)

**LT4 dose (μg/day)** *	500 (212.5–1112.5)

**LT4 dose (μg/kg/day)** *	27 (10.55–53.25)

**Symptoms**	33.3 (15)
Tachycardia	100.0 (15)
Hypertension	26.7 (4)
Agitation	20.0 (3)
Restlessness	6.7 (1)
Headache	6.7 (1)
Confusion	6.7 (1)

**TFTs**	
Normal	48.9 (22)
Abnormal	51.1 (23)

**Follow-up**	
Intensive care unit	17.8 (8)
Pediatric ward	28.9 (13)
Emergency department	53.3 (24)

**Treatment**	
Yes	55.6 (25)
No	44.4 (20)

**Treatment options**	
Activated charcoal	51.1 (23)
Gastric lavage	26.7 (12)
Propranolol	6.6 (3)
Methimazole	2.2 (1)
Methylprednisolone	2.2 (1)
Dexamethasone	2.2 (1)

**Hospital stay (hours)** *	24 (12–48)

**Outpatient follow-up duration (days)** *	3 (0–14)

**Time for TFT normalization (days)** *	0.5 (0–7)

* Data are presented as median (25th–75th percentile).

† p < 0.001 for age comparison between accidental and suicidal ingestion groups.

‡ p < 0.001 for comparison of multiple-drug ingestion between groups.

Abbreviations: n, number of individuals; LT4, levothyroxine; μg, microgram; kg, kilogram; TFTs, thyroid function tests.

**Table 2:** Detailed clinical characteristics of symptomatic patients (n = 15).

Age (years)	Cause	Time since ingestion (hour)	Multidrμg	Symptom onset	Symptom severity	Symptoms	LT4 dose (μg/day) (μg/kg/day)	TFTs	Treatment
2.8	Accidental	1–4	No	Acute	Moderate	Tachycardia Hypertension Agitation Restlessness	2100 (46)	Abnormal	Gastric lavage Activated charcoal Propranolol Methimazole Methylprednisolone
2.0	Accidental	1–4	No	Acute	Moderate	Tachycardia Agitation	1125 (75)	Abnormal	Activated charcoal Propranolol
4.0	Accidental	1–4	No	Delayed	Moderate	Tachycardia	600 (41)	Abnormal	Propranolol
3.4	Accidental	4–8	No	Acute	Mild	Tachycardia	4500 (281)	Abnormal	No
1.9	Accidental	1–4	No	Acute	Mild	Tachycardia	600 (30)	Abnormal	Activated charcoal
1.6	Accidental	0–1	No	Acute	Mild	Tachycardia	700 (53)	Normal	Activated charcoal
3.9	Accidental	1–4	No	Acute	Mild	Tachycardia Agitation	300 (18)	Normal	No
17.4	Suicidal	1–4	Yes	Acute	Moderate	Tachycardia Headache Confusion	7500 (136)	Abnormal	Gastric lavage Activated charcoal
15.0	Suicidal	1–4	Yes	Acute	Mild	Tachycardia	2500 (46)	Abnormal	Gastric lavage Activated charcoal
13.3	Suicidal	1–4	Yes	Acute	Mild	Tachycardia Hypertension	1100 (12)	Abnormal	No
14.0	Suicidal	1–4	Yes	Acute	Mild	Tachycardia	450 (9)	Normal	Activated charcoal Gastric lavage
16.5	Suicidal	0–1	Yes	Acute	Mild	Tachycardia Hypertension	375 (5)	Normal	Activated charcoal Gastric lavage
17.3	Suicidal	1–4	Yes	Acute	Mild	Tachycardia	225 (4)	Abnormal	No
15.0	Suicidal	1–4	No	Acute	Mild	Tachycardia Hypertension	2250 (35)	Abnormal	Activated charcoal
12.9	Suicidal	0–1	No	Acute	Mild	Tachycardia	1800 (20)	Abnormal	Activated charcoal Gastric lavage

Abbreviations: LT4, levothyroxine; μg, microgram; kg, kilogram; TFTs, thyroid function tests.

**Table 3:** Serum thyroid hormone levels at admission and follow-up.

	TSH (mIU/L)	fT4 (ng/dL)	fT3 (pg/mL)
**Admission (n=45)**	1.4 (0.9–2.1)	1.4 (1.1–2.9)	3.5 (3.1–4.8)
**12th hour (n=16)**	0.4 (0.2–1.8)	1.9 (1.4–2.9)	4.8 (3.5–6.0)
**24th hour (n=6)**	0.14 (0.04–0.34)	2.4 (1.9–5.3)	6.0 (4.5–9.0)
**48th hour (n=3)**	0.1 (0.1–0.1)	3.7 (2.7–4.0)	6.7 (3.5–9.4)
**60th hour (n=12)**	0.3 (0.0–1.4)	1.6 (1.2–2.4)	3.8 (3.3–6.0)
**72nd hour (n=6)**	0.0 (0.0–0.2)	2.0 (1.5–4.7)	5.4 (4.1–13.8)
**Week 1 (n=13)**	1.4 (0.3–1.9)	1.2 (1.1–1.9)	3.7 (3.2–5.0)
**Week 2 (n=13)**	1.5 (0.9–2.7)	1.3 (1.1–1.5)	3.6 (3.0–4.4)
**Week 4 (n=3)**	2.9 (0.8–4.8)	1.1 (1.0–1.4)	3.0 (3.0–3.0)

Data are presented as median (25th–75th percentile).

Abbreviations: TSH, thyroid-stimulating hormone; fT4, free thyroxine; fT3, free triiodothyronine; mIU, milliinternational units; L, liter; ng, nanogram; dL, deciliter; pg, picogram; mL, milliliter.

**Table 4:** Logistic regression analysis of factors associated with symptomatic presentation.

	B (SE)	Wald	p	Exp(B)	95% CI for Exp(B)	
					Lower	Upper
**LT4 Dose (μg/day)**	0.001 (0.001)	4.003	0.045	1.001	1.001	1.002
**Cause (suicidal)**	3.507 (1.216)	8.315	0.004	33.361	3.075	361.903
**Constant**	−0.871 (0.763)	1.302	0.254	0.419		

R^2^ = 0.40 (Cox and Snell), 0.57 (Nagelkerke); χ^2^ (6) = 2.93 (Hosmer and Lemeshow).

Abbreviations: B, regression coefficient; SE, standard error; Exp(B), exponentiated coefficient (odds ratio); CI, confidence interval; LT4, levothyroxine. A p < 0.05 was considered statistically significant.

## Footnotes

1 Medeiros-Neto G. Thyroxine poisoning. Endotext [online]. South Dartmouth (MA): MDText.com, Inc.; 2000–. Updated July 17, 2018. Website https://www.ncbi.nlm.nih.gov/books/NBK279036/ [accessed 21 December 2025].
